# Concentration dependent toxicokinetics of copper in the red flour beetle *Tribolium castaneum* (Coleoptera: Tenebrionidae)

**DOI:** 10.1007/s10646-015-1518-5

**Published:** 2015-07-14

**Authors:** Agnieszka J. Bednarska, Katarzyna Stępień

**Affiliations:** Institute of Environmental Sciences, Jagiellonian University, Gronostajowa 7, 30-387 Kraków, Poland

**Keywords:** Toxicokinetic, One-compartment model, Assimilation, Elimination, Respiration rate

## Abstract

To predict internal metal concentrations in animals under specific environmental exposures, the relationship between the exposure concentrations and values of toxicokinetic parameters must be known. At high exposure levels, the availability of carriers transporting metal ions through cellular membranes may become limited, thereby decreasing the assimilation rates (*k*_*A*_). Furthermore, increased metal concentrations in food may result in greater damage to the gut and reduce the assimilation efficiency and/or increase the elimination rate (*k*_*E*_). Therefore, *k*_*A*_ should decrease and *k*_*E*_ should increase with increasing metal concentrations. In fact, our study on *Tribolium castaneum* exposed to Cu at 500, 1000, 2000 and 4000 mg kg^−1^ of dry flour showed that with increasing Cu concentrations, *k*_*A*_ decreased from 0.0042 day^−1^ at 500 mg kg^−1^ to 0.0026 day^−1^ at 4000 mg kg^−1^ in females and from 0.0029 to 0.001 day^−1^ in males and *k*_*E*_ increased from 0.027 to 0.064 day^−1^ and from 0.018 to 0.04 day^−1^ in females and males, respectively. Significant differences in *k*_*A*_ between the sexes were observed at 2000 and 4000 mg kg^−1^, whereas significant differences between treatments were found for *k*_*A*_ in males. Copper was efficiently regulated by *T. castaneum*: an eightfold increase in exposure concentrations resulted in only a ca. twofold increase in the internal concentration. No Cu effect on the respiratory metabolism of *T. castaneum* was found.

## Introduction

Nutritional trace metals, such as Zn or Cu, are generally assumed to be efficiently regulated, whereas xenobiotics, such as Pb or Cd, are not (Spurgeon and Hopkin 1999). This difference should be reflected in distinct changes in the assimilation rate (*k*_*A*_) and/or the elimination rate (*k*_*E*_). Our recent study indicated that crickets, *Gryllus assimilis*, exposed to metals in food regulated their internal body concentrations of Zn by simultaneously changing their *k*_*A*_ and *k*_*E*_, whereas Cd was regulated almost exclusively through an increasing *k*_*E*_ (Bednarska et al. 2015). It is unknown whether other nutritional and xenobiotic metals are regulated in a similar manner. Moreover, the assimilation and elimination rate constants can be constant only under certain conditions, such as at a specific metal concentration. The higher the metal concentration in the environment, the higher the damage to the gut and the lower the assimilation efficiency and/or the higher the elimination rate (Argasinski et al. 2012). If the gut damage is occurring, then changes in the assimilation and/or eliminations rates of metals in animals fed metal-contaminated food or/and kept in a metal-contaminated medium should be observed according to changing metal exposures. The assimilation rates should decrease and the elimination rates should increase with increasing metal concentrations in food, and the magnitude of these effects should depend on the inherent metal toxicity. At high exposure concentrations, the availability of carriers by which metal ions are transported through membranes may also become limited, and thus, assimilation rates may decrease (Li et al. 2009).

Surprisingly, toxicokinetic studies have rarely been conducted at different metal exposure concentrations (He and Van Gestel 2013; Lock and Janssen 2001). However, to predict the internal metal concentrations in animals under specific environmental concentrations (pollution levels), the relationship between the exposure concentrations and values of toxicokinetic parameters must be known. Therefore, in this study, the red flour beetle, *Tribolium castaneum* (Coleoptera: Tenebrionidae), was exposed to copper at four different concentrations (500, 1000, 2000 and 4000 mg kg^−1^ dry flour) and the Cu toxicokinetic was followed over a 48-day long experiment.

Metals can affect the physiological traits of an organism even if it is able to immobilize and/or remove metals from the body (Stone et al. 2002). Because the processes of metal regulation in the body are energetically costly, we hypothesized that the increased cost of copper regulation and/or damage caused in the organism at higher concentrations should be reflected in increased respiratory metabolism in beetles unless irreversible pathological effects impair the metabolism itself (Calow 1989). Respiratory metabolism is one of the main components of an energy budget and is also relatively easy to measure. As such, it is often used as the equivalent of a metabolic rate (Handy and Depledge 1999; Migula 1989) and can serve as a convenient end-point in ecotoxicological studies if toxicant effects on the metabolic rate are expected (Laskowski et al. 1996; Migula 1989).

## Materials and methods

### Experimental design

*Tribolium castaneum* beetles were obtained from a stock culture kept at the Institute of Environmental Sciences, Jagiellonian University, in Krakow, Poland. The culture was established in 2009 from mixed populations obtained from different European laboratories and was maintained under standard laboratory conditions for this species: 30 °C, 70 % relative humidity (RH), darkness, in a medium of 90 % wheat flour and 10 % baker’s dried yeast. The flour was baked at 105 °C for 24 h to eliminate pathogens before mixing with yeast. Before starting the experiment, several hundred adult individuals were transferred to new boxes (20 individuals per box) with ca. 20 g of fresh medium and were left for 2 days to lay eggs. Next, the adults were sieved out and only the eggs were left in the medium until the pupal stage. At pupation, the beetles were separated by sex and the males and females were housed separately (ca. 20 individuals per box). At approximately 3 weeks after eclosion into adults, the beetles were placed individually in 1.5-ml Eppendorf-type tubes with perforated lids filled with ca. 1/2 of medium, either uncontaminated or contaminated with copper. Five treatments were prepared by adding water (control) or CuCl_2_*2H_2_O as an aqueous solution to obtain concentrations of 500, 1000, 2000 and 4000 mg Cu kg^−1^ dry medium. Next, flour was baked at 105 °C for 24 h, ground, sieved and mixed with ground yeast (9:1 dry weight). The nominal and actual concentrations of the metals in the medium in all of the treatments are listed in Table 1.

**Table 1:** Actual Cu concentrations in the medium (mean ± SD) and the estimated toxicokinetic parameters (*k*_*A*_ *—*assimilation rate constant, *k*_*E*_ *—*elimination rate constant) with asymptotic 95 % confidence intervals for the classic one-compartment model at four different concentrations for females (F) and males (M)

Cu concentration in medium (mg kg^−1^)		Sex	*C*_*I0*_ (mg kg^−1^)	*k*_*A*_ (day^−1^)	*k*_*E*_ (day^−1^)	*R* ^*2*^ (%)	*C*_*48*_ (mg kg^−1^)	*BAF*	*C*_*eq*_ (mg kg^−1^)
Nominal	Actual								
0	4.9 ± 0.13	F	18.3 ± 4.19	–	–	–	50 ± 13.1	–	–
		M	24.9 ± 8.47	–	–	–	36 ± 6.9	–	–
500	582 ± 27	F	18.3 ± 4.19	0.0042 (0.00268–0.0057)^A^	0.027 (0.0099–0.044)	0.0	45 ± 8.0	0.15	89.8
		M	24.9 ± 8.47	0.0029 (0.00167–0.0042)^a,A^	0.018 (0.0034–0.033)	0.0	43 ± 11.9	0.16	93.6
1000	1055 ± 6	F	18.3 ± 4.19	0.0026 (0.00189–0.0033)^A^	0.024 (0.0106–0.037)	0.0	53 ± 15.0	0.11	116.0
		M	24.9 ± 8.47	0.0017 (0.00097–0.0025)^a,A^	0.020 (0.0046–0.036)	0.0	46 ± 17.2	0.09	90.1
2000	1991 ± 43	F	18.3 ± 4.19	0.0022 (0.00153–0.0028)^A^	0.028 (0.0118–0.044)	9.3	70 ± 31.1	0.08	154.6
		M	24.9 ± 8.47	0.0011 (0.0007–0.00152)^b,B^	0.021 (0.0056–0.036)	0.0	50 ± 10.0	0.05	104.6
4000	4152 ± 74	F	18.3 ± 4.19	0.0026 (0.00149–0.0038)^A^	0.064 (0.0266–0.101)	20.8	84 ± 44.4	0.04	172.1
		M	24.9 ± 8.47	0.0011 (0.0007–0.00159)^b,B^	0.040 (0.0153–0.064)	18.5	50 ± 16.2	0.03	118.4

*C*_*I0*_ *—*internal metal concentrations in beetles at the start of the experiment; *R* ^*2*^ *—*determination coefficient of the fitted model; *C*_*48*_ *—*internal metal concentrations in beetles at the end of the experiment; *BAF—*bioaccumulation factor calculated based on the assimilation and elimination constants: *BAF* = *k*_*A*_ */k*_*E*_; *C*_*eq*_ *—*equilibrium concentration, i.e., the concentration expected in beetles at a specific external metal concentration in flour (*C*_*E*_) at t_∞_: *C*_*eq*_ = *C*_*E*_ *k*_*A*_ */k*_*E*_

Different lowercase letters mean significant differences (p ≤ 0.05) in *k*_*A*_ between concentrations for males

Different capital letters mean significant differences (p ≤ 0.05) in *k*_*A*_ between sexes for either 500, 1000, 2000 or 4000 mg kg^−1^ dry food

The beetles were randomly allocated to Cu treatments and were fed Cu-contaminated flour for 28 days (uptake phase). After 28 days, Cu-exposed beetles were transferred to uncontaminated flour (decontamination phase) for another 20 days. For chemical analyses, five males and five females from each Cu treatment were sampled randomly on days 0, 1, 2, 4, 6, 8, 12, 16, 20, 24, 28, 29, 30, 32, 33, 41 and 48. The control animals were kept in an uncontaminated medium throughout the experiment (48 days) and were monitored for background Cu levels in their bodies at days 4, 28 and 48. The sampled beetles were kept in an empty box for 24 h to empty the gut content, washed in deionized water to remove all remnants of flour from their body surface and killed by freezing at −20 °C. Animals that died over the course of the experiment were excluded from metal analysis.

### Respiration rate measurements

The respiration rate was measured on days 4, 28 and 48 in 50-ml glass bottles used as respiration chambers, with 9–10 individuals per bottle. The beetles (9–10 individuals together) were weighed 6 h before measurement. No food was offered during the respiration measurements, but to reduce desiccation, a 1.5-ml Eppendorf-type tube with a pierced lid, filled with distilled water, was placed in each bottle. Limited by the number of channels in the Micro-Oxymax respirometer (Columbus Instruments, USA), three replicates were conducted per treatment (0, 500, 1000, 2000 and 4000 mg kg^−1^ dry flour) per sex. The oxygen consumption was measured every 4 h for 28 h at 30 °C in darkness. The respiration rate was expressed in µl O_2_ mg^−1^ h^−1^. Prior to the data analysis, the first measurement point (the first 4-h interval) in each chamber was excluded from the data, as the change of the environment and handling stress could have temporarily caused abnormal activity and respiration rates in the beetles. The chambers where dead individuals were found after measurement were excluded from the statistical analysis of the respiration rate.

### Chemical analysis

The frozen beetles and medium (three samples per treatment) were dried at 105 °C for 24 h and were weighed to the nearest 0.001 mg (Radwag WPA 180/k, Radom, Poland) or 0.01 mg (Sartorius, Germany), respectively. The beetles were digested in 0.5 ml of a 1:4 mixture of perchloric (HClO_4_, 65 %, Ultranal; POCH, Poland) and nitric acids (HNO_3_, 65 %, INSTRA-Analysed; Baker, Germany) and were evaporated and resuspended to 0.7 ml with 0.2 % HNO_3_. Because of the large number of individuals, the beetles were analyzed in 6 series. The samples of the medium (ca. 0.5 g) were digested in 10 ml of a 1:7 mixture of perchloric and nitric acids and were then evaporated and resuspended to 50 ml with deionized H_2_O. The total concentrations of Cu in the beetles were measured with a graphite furnace atomic absorption spectrophotometer (Perkin-Elmer AAnalyst 800) and were measured in the medium with a flame atomic absorption spectrophotometer (Perkin-Elmer AAnalyst 200). To determine the analytical precision, three blanks and three samples of certified reference materials—fish liver (Dogfish Liver DOLT-4, National Research Council of Canada) and sea lettuce (*Ulva lactuca* No. 279, Institute for Reference Materials and Measurement, Belgium)—were run with the samples of either the beetles or the medium, respectively. The measured metal concentrations in the reference materials were within ±20 % of the certified values for all but one of the series. The results were not corrected for recovery. Instead, outliers (i.e., values that were more than 3.0 times the interquartile range above or below the upper or lower quartile) were excluded from further analysis. The results were expressed in mg kg^−1^ dry weight.

### Statistical analysis

The pattern of change in internal copper concentrations (*C*_*I*_) over time (*t*) was described with a one-compartment model (Skip et al. 2014):

$$ \begin{array}{ll} {\text{for}}\; t \leq t_c & C_{I} (t) = C_{I0} \cdot \,e^{{ - k_{E} \cdot \,t}} + C_{\text{Eu}} \frac{{k_{A} }}{{k_{E} }}(1 - e^{{ - k_{E} \cdot t}} ), \\ {\text{and}}\;{\text{for}}\; t>t_c & C_{I} (t) = C_{{It_{c} }} \cdot e^{{ - k_{E} \cdot (t - t_{c} )}} + C_{Ed} \frac{{k_{A} }}{{k_{E} }}\left( {1 - e^{{ - k_{E} (t - t_{c} )}} } \right),\\  {\text{where}} & C_{{It_{c} }} = C_{I0} \cdot e^{{ - k_{E} \cdot t_{c} }} + \, C_{Eu} \frac{{k_{A} }}{{k_{E} }}(1 - e^{{ - k_{E} \cdot t_{c} }} ). \end{array}$$

The symbols used in the model and the units of model parameters were as follows: *k*_*A*_*—*the assimilation rate constant (day^−1^), *k*_*E*_*—*the elimination rate constant (day^−1^), *t*_*c*_*—*the time of change in the medium from contaminated to uncontaminated (here: 28th day of the experiment) (days), *C*_*I*0_*—*the internal Cu concentration in the beetles at the start of the uptake phase (mg kg^−1^), and *C*_*Eu*_ and *C*_*E*d_*—*the Cu concentration in the medium (measured) in the uptake and decontamination phase, respectively (mg kg^−1^). The kinetic parameters *k*_*A*_ and *k*_*E*_ were obtained by fitting the abovementioned equations to the data from both experimental phases of each Cu treatment using the Marquardt method. The initial concentration, *C*_*I*0_, was explicitly given in the model as the average concentration of Cu measured in 19 females and 20 males before starting the experiment. All of the parameters were checked for significance using asymptotic 95 % confidence intervals. The asymptotic intervals around the estimated parameters were also used to compare the treatments and sexes. The bioaccumulation factor (BAF) was calculated based on the assimilation and elimination constants for the one-compartment model (*k*_*A*_/*k*_*E*_).

The distribution of Cu concentrations measured at the end of the experiment and the measured respiration rates were checked for normality with the Shapiro–Wilk’s *W* test, and the homogeneity of variances were checked with Levene’s test. One-way ANOVA was used to verify the ability of females and males to depurate down to the initial concentrations by comparing the Cu concentrations at days 0 and 48 for each Cu treatment. The Bonferroni correction for multiple comparisons was applied. Two-way ANOVA with treatment and sex as explanatory factors was used to check for differences in Cu concentrations at the end of the experiment. Furthermore, two-way ANOVA with day and sex as explanatory factors was used to check differences in Cu concentrations in the control beetles. The respiration rates were compared between the treatments, days of exposure and sexes using multifactor ANOVA.

All of the statistical analyses were performed using the Statgraphics Centurion XVI program (StatPoint Technologies, Inc., USA).

## Results

The measured Cu concentrations in the medium agreed with the nominal concentrations (Table 1). The measured Cu concentration in the uncontaminated medium was 4.95 (±0.13 SD) mg kg^−1^. Out of 1747 individuals used in both the toxicokinetic and respiration rate measurements, 48 died before the end of the experiment. In all of the treatments, mortality was below 4.0 %, indicating that even at 4000 mg Cu kg^−1^, Cu did not affect the beetles’ survival. Among the 698 values of body Cu concentrations, 22 values (less than 3 %) were excluded from the statistical analysis as outliers. Among the 90 measured respiration rates (3 runs, 30 chambers each), 2 cases were excluded from the statistical analysis due to the mortality of the beetles during measurement.

**Fig. 1:**
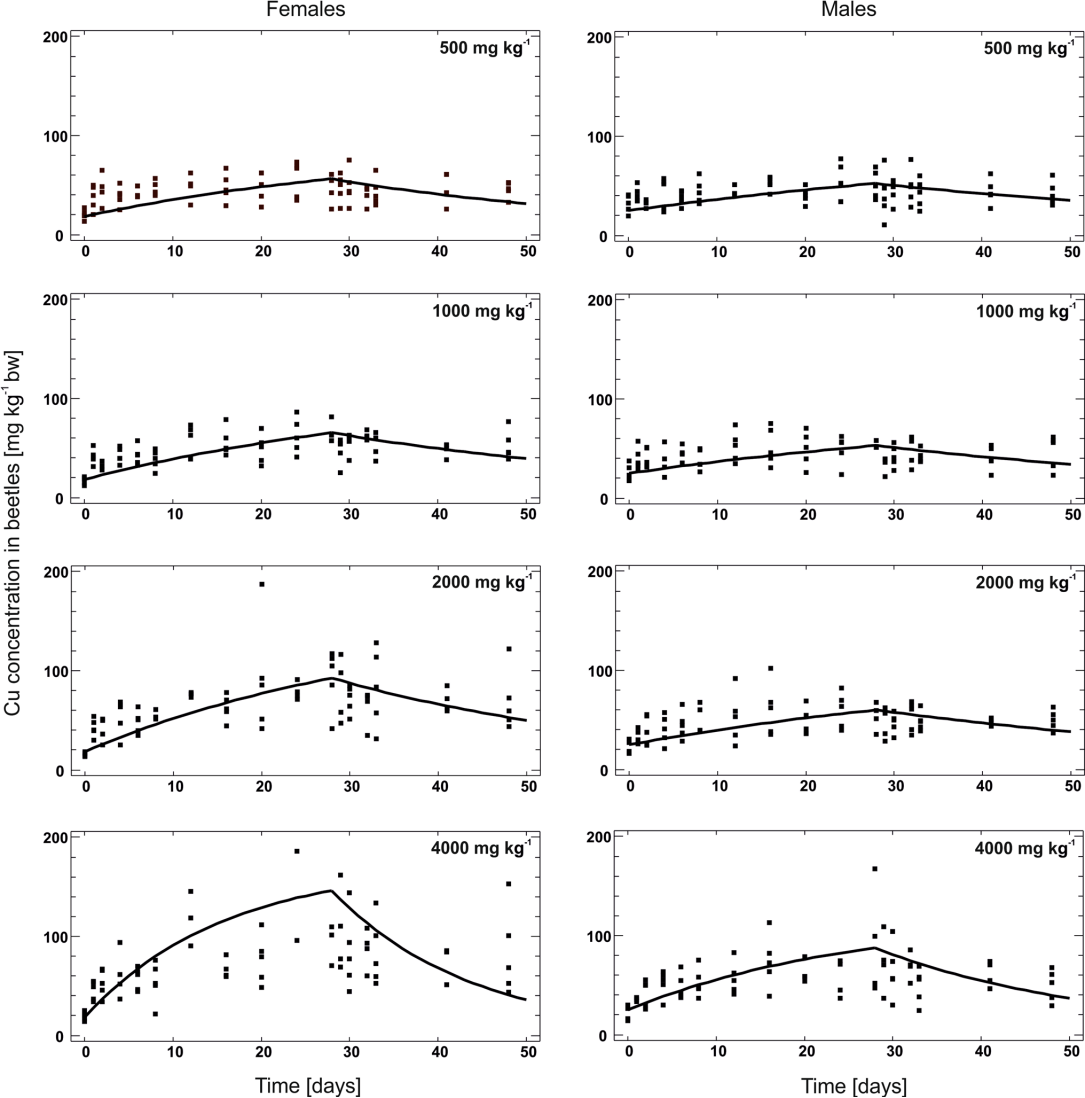
Copper kinetics in female (left-hand column) and male (right-hand column) red flour beetles, *Tribolium castaneum*, exposed to 500, 1000, 2000 and 4000 mg Cu kg^−1^ medium, as described by a one-compartment model. The *solid lines* indicate the fitted model. For the statistics for the parameter estimates, see Table 1

The internal Cu concentration in beetles at the start of the experiment (*C*_*I0*_) was lower in females (18.3 ± 4.19; mean ± SD) than males (24.9 ± 8.47 mg kg^−1^) (p = 0.004; *t* test). Although the Cu concentrations increased during the uptake phase in all treatments (Fig. 1), only at the two highest treatments did the model satisfactorily explain the variability in the Cu body concentrations (Table 1). Nevertheless, the estimated kinetics parameters were significant in all of the treatments: the asymptotic 95 % confidence intervals did not cover 0 in any cases.

**Fig. 2:**
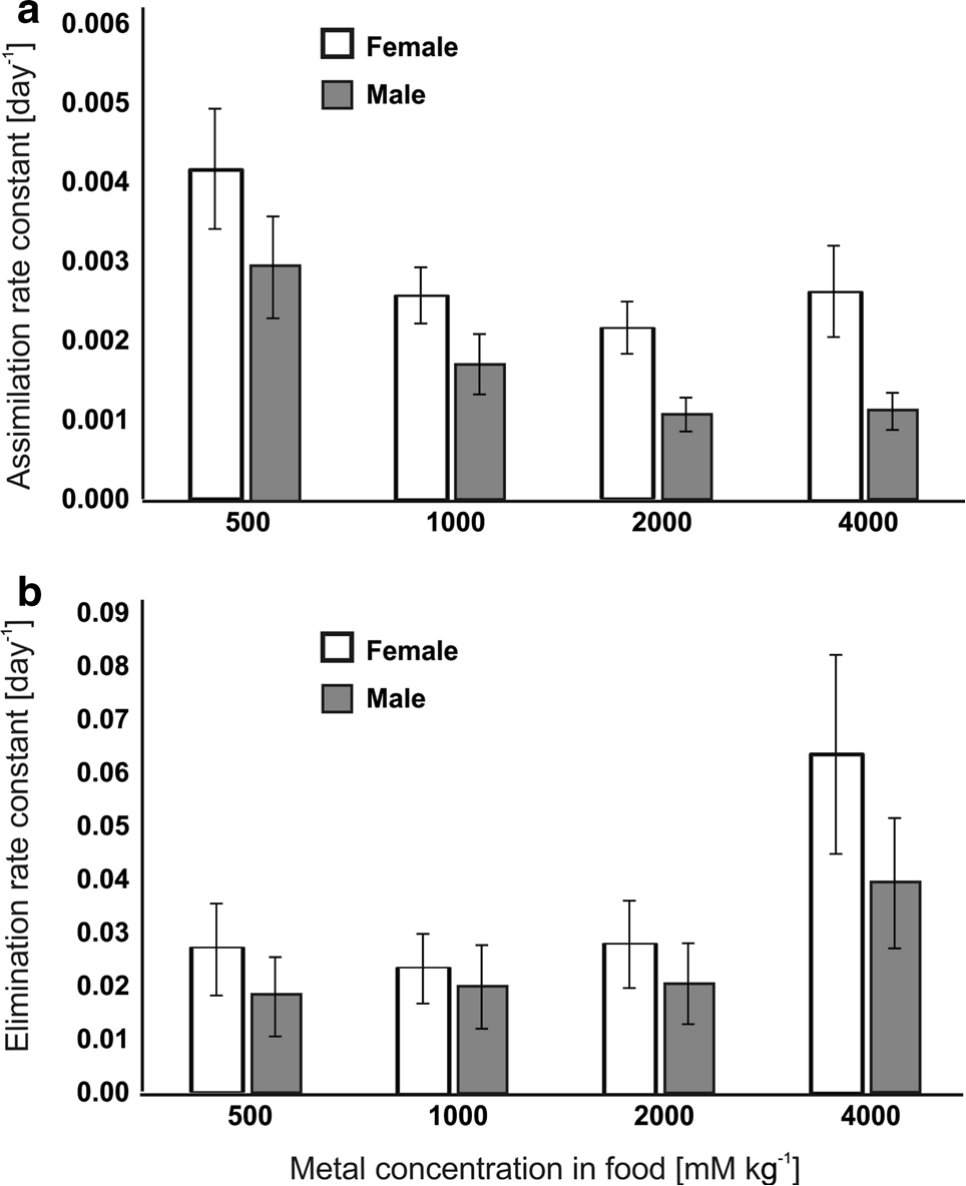
Assimilation rate constants, *k*_*A*_ (**a**) and elimination rate constants, *k*_*E*_ (**b**) for *Tribolium castaneum* exposed to a 500, 1000, 2000 and 4000 mg Cu kg^−1^ medium; whiskers indicate standard error of the *k*_*A*_ and *k*_*E*_

With increasing Cu concentration in the medium, the *k*_*A*_ decreased both in females and males (Fig. 2a). Significant differences in *k*_*A*_ were found in males exposed to 500 and either 2000 or 4000 mg Cu kg^−1^. Females exposed to Cu at concentrations of 2000 and 4000 mg kg^−1^ had a significantly higher *k*_*A*_ than males originating from those treatments (Table 1). In females, the *k*_*E*_ was the lowest at 1000 mg Cu kg^−1^ and the highest at 4000 mg Cu kg^−1^. In males, the *k*_*E*_ increased with increasing Cu concentrations in the medium (Fig. 2b). The large variance in the internal body concentrations between individuals and the resulting broad confidence intervals (Table 1) did not allow for detecting significant differences in *k*_*E*_ between the treatments or sexes. Our results indicated that the body concentration of Cu was maintained by simultaneously changing *k*_*A*_ and *k*_*E*_, and an eightfold increase in the exposure concentration (from 500 to 4000 mg kg^−1^) resulted in less than a twofold increase in the internal concentration (Table 1). The estimated equilibrium concentration (*C*_*eq*_ = *C*_*E*_*k*_*A*_*/k*_*E*_) of Cu in beetles increased with increasing Cu concentrations in the medium, but more substantially in females than in males (Table 1).

The internal Cu concentration was generally higher at the end of the elimination phase than at the beginning of the uptake phase in all of the treatments (p ≤ 0.001 for males, p ≤ 0.0001 for females) (Table 1). After applying the Bonferroni correction for multiple comparisons, only the difference in the control males appeared to be nonsignificant. A trend of increasing internal Cu concentrations measured on the 48th day of the experiment with increasing Cu exposure was observed; females accumulated more Cu than males (p = 0.012). Females accumulated up to 80 mg Cu kg^−1^ at the highest treatment, whereas males accumulated between 43 mg Cu kg^−1^ at 500 mg kg^−1^ and 50 mg Cu kg^−1^ at 4000 mg Cu kg^−1^, but the treatment effect was barely significant (p = 0.06) (Table 1). The Cu concentrations in the control beetles monitored on days 4, 28 and 48 were above the levels at the beginning of the experiment and increased over time (p = 0.008), which was especially clear for females that had a higher Cu concentration than males (p = 0.04). The mean (±SD) Cu concentrations on days 4, 28 and 48 were as follows: 27.2 ± 3.48, 41.3 ± 11.25 and 50.4 ± 13.1 mg kg^−1^ in the control females and 24.8 ± 7.31, 32.7 ± 15.41 and 36.3 ± 6.93 mg kg^−1^ in the control males.

**Fig. 3:**
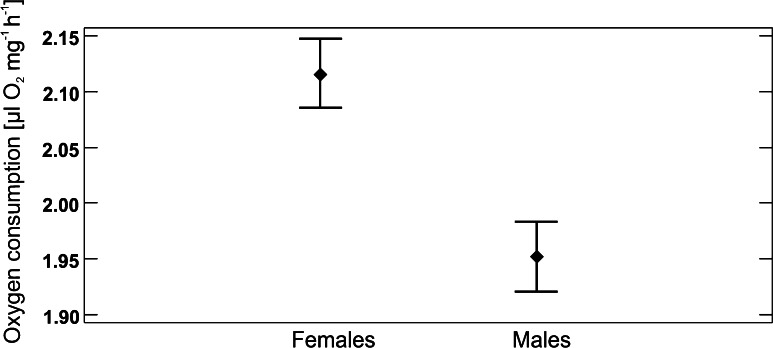
Mean respiration rates in female and male *Tribolium castaneum* adjusted for the effect of other variables in the model; differences between the sexes were significant at p ≤ 0.0001. Another variables in the model were the day of the experiment (p ≤ 0.0001) and Cu treatment (p > 0.9); whiskers indicate 95 % LSD intervals

No Cu effect on oxygen consumption was found (p > 0.9). Only the sex and time affected the respiration rates (p ≤ 0.0001): the females had higher respiration rates than the males (Fig. 3) and the respiration rates were significantly higher at the end of the experiment than on days 4 and 28, which did not differ between each other.

## Discussion

Although past measurements of metal body concentrations in animals collected from contaminated areas indicated that metals are regulated by exposed individuals to some extent, the process required verification in laboratory experiments. In this study, we used different copper concentrations in an exposure medium (flour) to directly compare the values for kinetic parameters between different concentrations. In this way, we were able to show that internal Cu concentration in *T. castaneum* was maintained through a simultaneous decrease in the assimilation rate (*k*_*A*_) and an increasing elimination rate (*k*_*E*_). The changes in toxicokinetic parameters with increasing exposure were not reflected in the respiratory metabolism of *T. castaneum*.

The uptake rate (i.e., the amount of substance ingested by an organism per unit time) has been suggested as a better indicator of sublethal toxicity than the internal metal concentration itself. For example, Van Straalen et al. (2005) indicated how sub-lethal effects of Zn on the isopod *Porcelio scaber* depended on the rate at which Zn entered the organism. Furthermore, the rate of metal assimilation and/or elimination can be constant only under certain conditions, such as at a specific metal concentration (Bednarska et al. 2015, Lock and Janssen 2001). The mechanisms of the internal regulation of metals by invertebrates have seldom been formally tested, especially in terms of the possible metal accumulation strategy of invertebrates. He and Van Gestel (2013), who exposed the potworm *Enchytraeus crypticus* to six different Ni concentrations in quartz sand, indicated that the assimilation rate constant increased when the exposure concentration increased up to the middle of the studied range (0.053–0.94 mg Ni l^−1^) and then decreased. The *k*_*E*_ values were almost constant, except for the lowest exposure concentration at which the *k*_*E*_ was an order of magnitude lower than at other concentrations (He and Van Gestel 2013). In a study on the uptake and elimination kinetics of Zn and Cd in oligochaetes, Lock and Janssen (2001) found that the assimilation rate constant of Cd in *Enchytraeus albidus* decreased by a factor of two when increasing the exposure concentration from 10 to 100 mg kg^−1^ dry weight of soil. However, the authors could not calculate the kinetic parameters in Zn for comparison between the exposure concentrations due to the fluctuations in the internal metal concentrations during the uptake period. Our previous study on orally exposed crickets showed that the body concentration of Zn was regulated by simultaneously changing the assimilation and elimination rates, whereas the Cd concentration was regulated almost exclusively through an increased elimination rate (Bednarska et al. 2015). This study suggests that Cu is regulated by *T. castaneum* in a similar manner to Zn by crickets and in an efficient way; an eightfold increase in the Cu exposure concentration (this study) and 16-fold increase in the Zn exposure concentration (Bednarska et al.^2015^) resulted in only ca. twofold increase in the internal metal concentration. Also, *Folsomia candida* appeared to limit the accumulation of copper at both high (100 mg kg^−1^ soil) and low (25 mg kg^−1^ soil) exposure levels but was mostly due to the decreasing *k*_*A*_ with an increasing Cu exposure concentration (Ardestani and Van Gestel 2013).

If metals are detoxified by excretion, the body concentrations should decrease when previously exposed individuals are transferred to a clean environment. However, if an element is bound to an inorganic matrix or to organic ligands, the metal levels may remain constant, even after exposure has ceased (Spurgeon and Hopkin 1999). The higher internal Cu concentration in *T. castaneum* at the end of the decontamination phase compared to the levels before the start of the uptake phase can be attributed to the detoxification of copper inside the body, which is partly due to storage. Similar results were found for Cu exposed *F. candida* (Ardestani and Van Gestel 2013), but not for earthworms, *Eisenia fetida* (Spurgeon and Hopkin 1999), in which Cu was detoxified primarily by excretion. Nevertheless, the concentration of Cu in *T. castaneum* dropped significantly during the decontamination phase, indicating significant Cu elimination from the body, as reflected by significant *k*_*E*_ values in all of the treatments.

Copper has an important role in the structure and function of enzymes and proteins. Like many other metals (e.g., Ni, Zn, Fe), Cu is an essential element for many species and either deficiency or toxicity symptoms may occur when too little or too much Cu is assimilated. In our study, even in the control beetles kept in the flour with only a background Cu concentration (ca. 5 mg kg^−1^), there was some increase in the internal Cu concentration over time. Such results may indicate that the beetles were actively assimilating Cu from the control medium to the levels needed for proper metabolic activity. The concentration of ca. 5 mg Cu kg^−1^ flour used in our control medium was likely below the optimal concentration for *T. castaneum,* which was assessed by Medici and Taylor (1966) as ca. 13 mg Cu kg^−1^ flour. The normal body Cu concentrations are apparently different for males and females. Although females had significantly lower body Cu concentrations than males at the beginning of the experiment, they accumulated Cu from the control medium up to a concentration of ca. 1.4-fold higher than males after 48 days of the experiment. The relatively high Cu concentration in the control females after 48 days, of ca. 50 mg Cu kg^−1^ dry body weight, is still within the range found for other species from unpolluted environments. For example, Warchałowska-Śliwa et al. (2005) found that orthopteran *Tetrix tenuicornis* from areas with a background Cu level of 3.5–9 mg kg^−1^ soil accumulated 50–110 mg Cu kg^−1^ dry body weight.

The maintaining of internal Cu concentrations in Cu exposed beetles on relatively low level was not reflected in their metabolic rate. This result may suggest that the beetles did not suffer serious damage to the gut, as such damages should be visible in the metabolic rate (Laskowski et al. 1996). Previously, elevated respiration rates were observed in laboratory strains of confused flour beetles (*Tribolium confusum*) that were treated for several generations with copper (Lukasik and Laskowski 2007). The opposite reaction—a decrease in the respiration rates—was observed in centipedes treated with 640 mg Cu kg^−1^ food, but this effect was found after short exposures only and was not retained during longer-term exposure (Laskowski et al. 1996). Similarly, a decrease in respiratory metabolism was found in house crickets (*Acheta domesticus*) intoxicated with cadmium, while neither zinc nor lead affected their respiration rate (Migula 1989). The abovementioned studies emphasize the importance of exposure concentrations and the time of exposure to different metals to induce the observed effects on respiration rates. Different mechanisms may be activated during exposure to different metals, and these are not always reflected in the respiration rates. In general, the oxygen consumption rate measured in *T. castaneum* in our study was in the range of 1.82–2.12 µl O_2_ mg^−1^ h^−1^, which is similar to the value of 2.97 µl O_2_ mg^−1^ h^−1^ found in *T. castaneum* at 30 °C by Emekci et al. (2002). Our results also fall within the range of 1.79–2.31 µl O_2_ mg^−1^ h^−1^ reported by Park (1936) for a similar species, *T. confusum*, at 28 °C. Moreover, the sex-specific metabolism that we identified in this study was previously reported in confused flour beetle, *T. confusum* (Lukasik and Laskowski 2007).

In conclusion, internal copper level was maintained by *T. castaneum* through a simultaneous decreasing assimilation rate and increasing elimination rate and this regulation of Cu did not incur energetic costs in terms of the respiratory metabolism of *T. castaneum*.
